# Mg/Mn co-doped CdS nanoparticles: a multifunctional platform for wastewater purification and antibacterial applications

**DOI:** 10.1039/d6ra00066e

**Published:** 2026-04-21

**Authors:** Tahir Iqbal, Muhammad Faisal Ayaz, Sumera Afsheen, Mohsin Ijaz, Yasir Hussain, Muhammad Yousaf, Muhammad Shehzad Sultan, Jawza A. Almutairi, Nabil Al-Zaqri

**Affiliations:** a Department of Physics, Faculty of Science, University of Gujrat Hafiz Hayat Campus Gujrat 50700 Pakistan tahir.awan@uog.edu.pk; b Department of Zoology, Faculty of Science, University of Gujrat Hafiz Hayat Campus Gujrat 50700 Pakistan; c Department of Physics, University of Otago Dunedin 9054 New Zealand mohsin.ijaz@external.otago.ac.nz; d Department of Mechanical Engineering, Faculty of Engineering, University of Gujrat Hafiz Hayat Campus Gujrat 50700 Pakistan; e Department of Physics, University of Puerto Rico-Rio Piedras San Juan PR 00925 USA; f Department of Pharmaceutical Sciences, College of Pharmacy, Princess Nourah bint Abdulrahman University P.O. Box 84428 Riyadh 11671 Saudi Arabia; g Department of Chemistry, College of Science, King Saud University P.O. Box 2455 Riyadh 11451 Saudi Arabia nalzaqri@ksu.edu.sa

## Abstract

Developing nanomaterials with multifunctional properties that can address both biomedical and environmental challenges is the focus area of this research study. This work presents Mg and Mn co-doped CdS synthesized in a controlled way by a co-precipitation method utilizing ammonia hydroxide as both a capping and stabilizing agent. While fixing the Mn concentration at 2%, the Mg concentration was varied from 1% to 7% based on prior studies of the optical and electronic properties of CdS at these doping concentrations. X-ray diffraction analysis confirmed lattice expansion and a hexagonal phase along with crystal size reduction from 49.08 nm (pure CdS) to 42.94 nm for Mg_5%_–Mn_2%_-CdS, which is evidence of successful substitution. The reduction in the optical bandgap from 2.80 eV (pure CdS) to 2.19 eV (optimally co-doped sample) is evidence of the absorption enhancement of visible light. Methylene blue exhibited 84.79% degradation under visible-light irradiation within 125 min, following psuedo-first-order kinetics with a rate constant of 0.014 min^−1^ (*R*^2^ = 0.98). The dominant reducing species, ˙O_2_^−^ and ˙OH, were confirmed by scavenger experiments. The same sample exhibited strong antibacterial activity by producing inhibition zones of 17 ± 0.2 mm against *E. coli* and 40 ± 0.3 mm against *S. aureus*, which surpassed the activity of pure CdS, approaching the standard antibiotic efficiency. These results suggest that Mg–Mn-co-doped samples are stable, dual-functional, and suitable for both wastewater purification and antimicrobial applications.

## Abstract 2

Developing nanomaterials with multifunctional properties that can address both biomedical and environmental challenges is the focus area of this research study.

## Graphical abstract

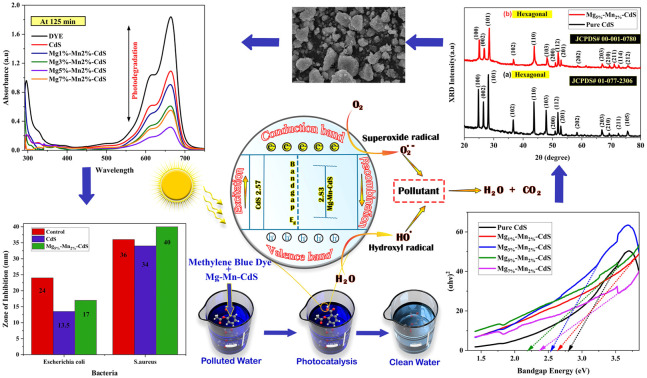

## 1. Introduction

Water bodies contaminated by the discharge of organic dyes and pathogenic microorganisms are a serious threat to public health and environmental sustainability.^1^ The primary sources of these organic pollutants involve industrial effluents, mainly from textile, pharmaceutical and dyeing industries, which discharge large quantities of non-biodegradable, toxic and carcinogenic synthetic dyes.^2^ Moreover, the increasing prevalence of antibiotic-resistant bacteria, such as *Escherichia coli* and *Staphylococcus aureus*, further complicates efforts to ensure the availability of safe and clean water resources.

The problem of removing dyes from aqueous media has been addressed by various methods, but not all of them are effective. Therefore, researchers have explored several physical, chemical and biological approaches that can eliminate inorganic and organic contaminants from water. Some of these techniques include adsorption, advanced oxidation processes, photocatalytic degradation,^3^ reverse osmosis, bio-degradation^4^ and ultrafiltration.^5^ However, photocatalytic degradation has garnered considerable interest in the field of pollutant degradation because of its environmental benefits and capacity to degrade different pollutants.^6–8^ In addition to pollutant degradation, antimicrobial activity plays a crucial role in wastewater treatment. Pathogens such as *E. coli* and *S. aureus* can be eliminated with materials possessing strong antibacterial properties. Nanoparticles can inactivate these microorganisms by disrupting their bacterial membranes and interfering with their cellular metabolism by introducing oxidative stress.^9^ Among the various assessment methods for the antimicrobial activity, which include the agar well diffusion method, disc diffusion method, broth dilution method, time-kill assay, and agar dilution method, the disc diffusion method is most suitable because of its simplicity, reproducibility, and ease of comparing inhibition zones between samples and standard antibiotics.^10^

Semiconductor photocatalysis has been intensively explored for its extensive amplification of environmental contamination.^9^ Amicable semiconductors like WO_3_, TiO_2_, Cu_2_O, Fe_2_O_3_, PbS, CdS, ZnS, SnO_2_, ZnO, and Bi_2_WO_6_ can be used as photocatalysts for photocatalytic degradation purposes because of their appropriate band gap energies.^11^ Cadmium sulphide (CdS) is one of the best catalysts that has gained considerable attention due to its remarkable optical and electrical properties, leading to its widespread application in solar energy conversion, photoelectronic devices and photocatalytic degradation.^12,13^ Cadmium sulphide is the most studied photocatalytic semiconductor, possessing a bandgap of 2.4 eV, which makes it a suitable option for the application of photocatalysts because of its absorption of visible light. Nevertheless, the fast recombination of photogenerated electron–hole pairs limits the photocatalytic efficiency of CdS. The most important challenge for CdS photocatalysts is their instability at elevated temperatures as performance is lowered due to sintering. Recent studies show that controlling particle geometry (as with the ex-solution strategy on perovskite oxide supports) can enhance stability and catalytic efficiency.^14^ Doping has become a focal point of research for enhancing the performance of CdS because it can change the electronic structure of the material and can enhance charge separation.

The intrinsic band structure of semiconductors can be tuned to improve visible-light sensitivity and their photocatalytic activity.^15^ The motivation for our research stems from the fact that several metal doping ions, including iron (Fe), manganese (Mn), magnesium (Mg), silver (Ag), and copper (Cu), have been shown to reduce the band gap and improve light absorption characteristics.^16^

In countries with limited resources, textile wastewater containing dyes is often reused for agricultural hydration. A significant amount of these dyes ends up in the soil, especially in areas close to textile manufacturing facilities.^17^ This is because the textile industry uses enormously toxic synthetic dyes that are physically and chemically stable. It creates significant risk for the environment. Synthetic dyes are extremely harmful to the environment because of their persistence and accumulation in the soil.^18^ The degradation of harmful products has been reported many times using CdS, along with its electrochemical properties. However, there are no reports of magnesium and manganese co-doping occurring in the same instant. Co-doping has been shown to improve several aspects, such as optical absorbance and photocatalytic capabilities, compared with the original CdS material.

Co-doping with Mg^2+^ (ionic radius = 0.72 Å) and Mn^2+^ (ionic radius = 0.80 Å) is an effective way for controlling the structural and electronic properties of CdS. Mg^2+^ leads to lattice contraction and strain, whereas Mn^2+^ introduces electronic states near the band edges that change light absorption and recombination.^19,20^ Their combination forms a state of synergistic balance, which increases optical absorption, suppresses radiative recombination and changes surface reactivity. This paper pays attention to learning the structure–property relation of Mg/Mn co-doping, which is not directly concerned with the photocatalytic performance.^21,22^

According to a literature review, Mn-doped CdS introduces magnetic properties and extends the adsorption spectrum to enhance light absorption and charge separation for improved photocatalysis.^23^ In contrast, Mg-doped CdS increases the photodegradation of organic compounds by improving light absorption and charge carrier dynamics, resulting in more effective pollutant breakdown.^24^ So, we decided to add manganese to each sample at a fixed concentration of 2% by weight^25^ while varying the concentrations of magnesium at 1%, 3%, 5%, and 7% by weight to increase pollutant removal capabilities.

This research work is novel because of the first-time synthesis of Mg–Mn-co-doped CdS nanoparticles utilizing a reliable co-precipitation method for synthesis purpose, with ammonium hydroxide used as a capping as well as stabilizing agent. Co-doping not only modifies the electronic band structure and narrows the band gap but also significantly reduces electron–hole recombination and enhances visible-light response. The optimal composition, Mg_5%_–Mn_2%_-CdS, exhibited higher photocatalytic degradation efficiency against methylene blue dye and demonstrated improved antibacterial activity against *Escherichia coli* and *Staphylococcus aureus*. This dual performance marks the potential of the material as a multifunctional agent for wastewater treatment and antimicrobial applications.

## 2. Experimental methodology

### 2.1 Materials

The reagents, which are utilized for the synthesis of pure CdS and Mn–Mg-co-doped CdS nanostructures *via* the coprecipitation method, include cadmium chloride (CdCl_2_) and sodium sulphide (Na_2_S), along with ammonium hydroxide solution (NH_4_OH) as both a capping and stabilizing agent. Magnesium chloride hexahydrate and manganese(ii) chloride tetrahydrate are used as the sources of Mg and Mn, respectively. All chemicals were obtained from Sigma-Aldrich with a purity level of 99.98% and used without additional purification processes for their reliability and consistency in supporting the scientific objectives.

### 2.2 Synthesis of the photocatalyst

The chemical precipitation method was used to synthesize the pure CdS. First, 0.05 moles of CdCl_2_ (9.17 g) were dissolved in 50 mL of distilled water, and 0.05 moles of Na_2_S (3.92 g) were dissolved in 30 mL of distilled water. These solutions were subjected to ultrasonication for 40 minutes to attain a uniform solution at a temperature of 80 °C. After this sulphide source solution, Na_2_S was gradually added to the hot cadmium salt solution (CdCl_2_) under constant sonication for 30 minutes. As a result, the precipitate of CdS was formed immediately and started settling down. Ammonium hydroxide was added dropwise to the mixture while stirring to maintain the pH between 9 and 10 to prevent the formation of Cd(OH)_2_ and control particle size. The precipitate was washed numerous times with distilled water and ethanol, then dried in an oven at 80 °C for 5 hours. Finally, the obtained material was calcined at 450 °C for 6 hours to improve its morphology and crystallinity. The obtained powder was manually ground with a mortar and pestle for ∼25–30 minutes to obtain fine nanoparticles.

**Fig. 1:**
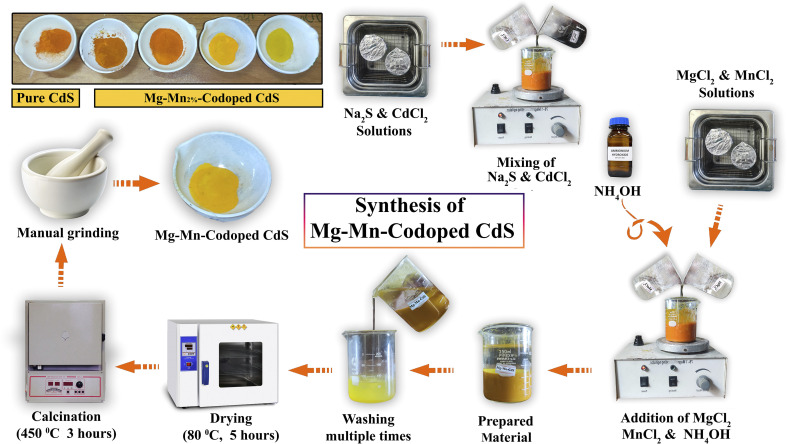
Synthesis of pure CdS and Mg–Mn-CdS by the coprecipitation method.

In the case of Mn–Mg-co-doped CdS NPs, two distinct solutions were prepared separately using ultrasonication. The first solution was made by dissolving magnesium chloride hexahydrate (MgCl_2_·6H_2_O) in distilled water. The other solution was made by dissolving MnCl_2_·4H_2_O in distilled water, as indicated in Fig. 1. The CdCl_2_ precursor solution was prepared in stoichiometric amounts corresponding to the desired Mn (2%) and Mg (1%, 3%, 5%, and 7%) doping levels for CdS synthesis. The next procedure was the same as for the synthesis of pure CdS, and after manual crushing, a yellow powder of Mn–Mg-CdS was obtained.

## 3. Characterization

Various characterization techniques were used to analyze the unique properties of Mn–Mg-co-doped CdS. XRD analysis was used to assess the purity and crystallinity, and SEM analysis to examine the surface morphology and its correlation with photocatalysts. Energy-dispersive X-ray spectroscopy (EDX) analysis was used to confirm the elemental composition of Cd, S, Mg and Mn in the samples. It is observed that EDX analysis is a semi-quantitative compositional technique, especially for light elements, and hence, the concentrations of dopants reported represent the nominal values used during synthesis. The main goal of EDX analysis in this study is to verify the existence and homogeneity of Mg and Mn dopants and not to provide absolute bulk quantification. Furthermore, photoluminescence (PL) analysis was used to investigate the existence of defect states and charge carrier behavior, which influence the photocatalytic performance of the material. FTIR analysis was used to identify the existence of functional groups. The band gap variations of the Mg–Mn-CdS samples with different compositions were detected by UV-vis spectroscopy analysis, which shows an impact on photovoltaic performance. Eventually, the photocatalytic activity was evaluated to verify that Mn–Mg-co-doped CdS nanomaterials exhibit better photocatalytic activity than pure CdS nanoparticles.

### 3.1 Structural and crystallographic analysis

X-ray diffraction (XRD) analysis was applied to determine the structural and phase composition of pure CdS and Mg_*X*_–Mn_2%_-co-doped CdS nanomaterials, where *X* = 1%, 3%, 5%, and 7%. The patterns obtained through diffraction were matched JCPDS reference cards in order to determine the crystal structures, phase purity, and lattice parameters.

In the XRD analysis of the pure and doped variants of CdS samples, both samples were found to exhibit the same hexagonal crystal form with a space group of *P*6_3_*mc*.^26^ For the pure CdS sample, the lattice parameters were *a* = *b* = 4.136 Å, and *c* = 6.713 Å, with angles *α* = *β* = 90° and *γ* = 120°, and the calculated cell volume and theoretical density of pure CdS were 99.45 × 10^6^ Å^3^ and 4.82 g cm^−3^, respectively. The diffraction peaks at angles of 24.8°, 26.5°, 28.2°, 36.7°, 43.7°, 47.8°, and 51.8° correspond to the crystal planes (100), (002), (101), (102), (110), (103), and (200), respectively.^27^ The highest peak values for 2*θ* were assigned to the (100), (002), and (101) planes, which were consistent with the hexagonal phase of CdS.

**Fig. 2:**
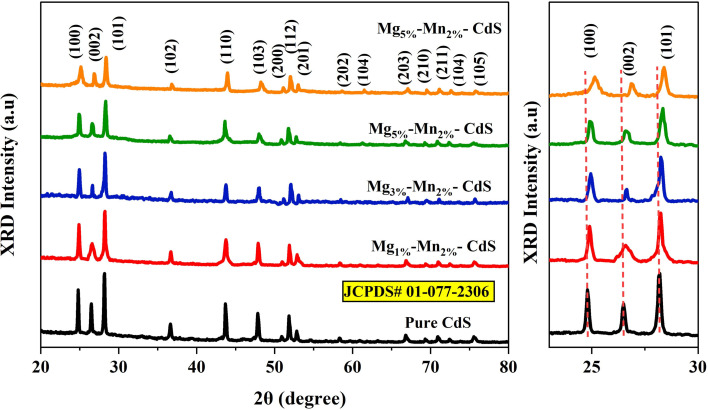
(Left) XRD spectra of pure CdS and Mg_1%_–Mn_2%_-CdS, Mg_3%_–Mn_2%_-CdS, Mg_5%_–Mn_2%_-CdS and Mg_7%_–Mn_2%_-CdS nanoparticles. (Right) Enlarged view of the XRD peaks corresponding to the (100), (002) and (101) crystal planes.

All doped samples exhibited the same diffraction peaks, corroborating the existence of a hexagonal structure. A slight increase in lattice parameters and cell volume was observed with increasing doping concentration, suggesting that the lattice expanded due to the incorporation of Mg^2+^ and Mn^2+^ ions. The crystallite size was calculated for all samples using the Debye–Scherrer formula. A slight shift of the major diffraction peaks towards higher angles indicated that there was a slight alteration in the lattice, which may have been brought about by the decrease in crystallite size and the lattice strain introduced by Mg^2+^ and Mn^2+^ sourced in place of Cd^2+^, as shown in Fig. 2(a). The broadening of the (002) reflection, especially at low Mg content, is attributed to the presence of dopant-induced microstrain and crystallite size changes due to the ionic radius mismatch between Cd^2+^ and the substituted Mg^2+^/Mn^2+^ ions. The absence of additional phases confirms successful doping.^28^

The Debye–Scherrer formula was used to estimate the average crystallite size as follows:1
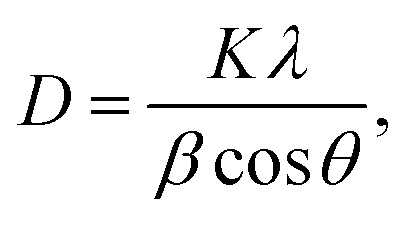
where *D* is the average crystallite size, *k* = 0.96 is the shape factor, *λ* = 0.15406 nm is the X-ray wavelength, *β* is the full width at half maximum (FWHM), and *θ* is the Bragg diffraction angle.

The calculated crystallite size of the pure CdS sample was about 4.9083 × 10^−8^ m (49.8 nm), whereas the Mg_5%_–Mn_2%_-CdS doped sample showed a reduced crystallite size of 4.294 × 10^−8^ m (42.94 nm). The broadening of the (002) diffraction peak, especially for the Mg_1%_–Mn_2%_-CdS sample, can be attributed to a combination of decreased crystallite size and increased microstrain caused by the incorporation of the dopants. The substitution of smaller Mg^2+^ (0.72 A) and Mn^2+^ (0.80 A) ions at Cd^2+^ (0.97 A) lattice positions causes local lattice distortion and strain, which is responsible for peak broadening, as is consistent with XRD line-broadening theory. Additionally, reduced concentrations of dopant may cause a non-uniform concentration of dopant, further increasing strain-related broadening effects. Similar broadening of the (002) reflection due to the presence of dopants has been widely reported in metal-doped CdS and related II–VI semiconductor systems. The reduction in crystallite size caused by doping confirms better nucleation and finer grain formation, which helps improve optical absorption and photocatalytic performance due to the increased surface area in this study.^29^ Analysis of these results indicates that the Co-doping of Mg and Mn effectively alters the crystal structure, enlarges the lattice volumes, reduces the crystallite size, and enhances the performance of the material for potential photocatalytic and antimicrobial applications.

The slight variation in peak intensity and broadening in these samples suggests that higher Mg doping favors lattice disorder and reduces crystallite size, as shown in Table S1. Structural parameters obtained from XRD analysis show a small decrease in average crystallite size for the Mg_7%_–Mn_2%_-CdS sample, which leads to the assumption that the size effect contributes to peak broadening in the sample. The values of microstrain provide further evidence that the lattice distortion caused by the dopants contributes to the observed line broadening. Given the similarities in valence and ionic radii, Mg^2+^ and Mn^2+^ are likely to occupy Cd^2+^ substitutional sites and not interstitial sites within the CdS structure. The calculated lattice parameters indicate a slight decrease with increasing dopant concentration, supporting a contraction of the lattice by the incorporation of smaller Mg^2+^ and Mn^2+^ ions.

### 3.2 SEM image analysis and discussion

**Fig. 3:**
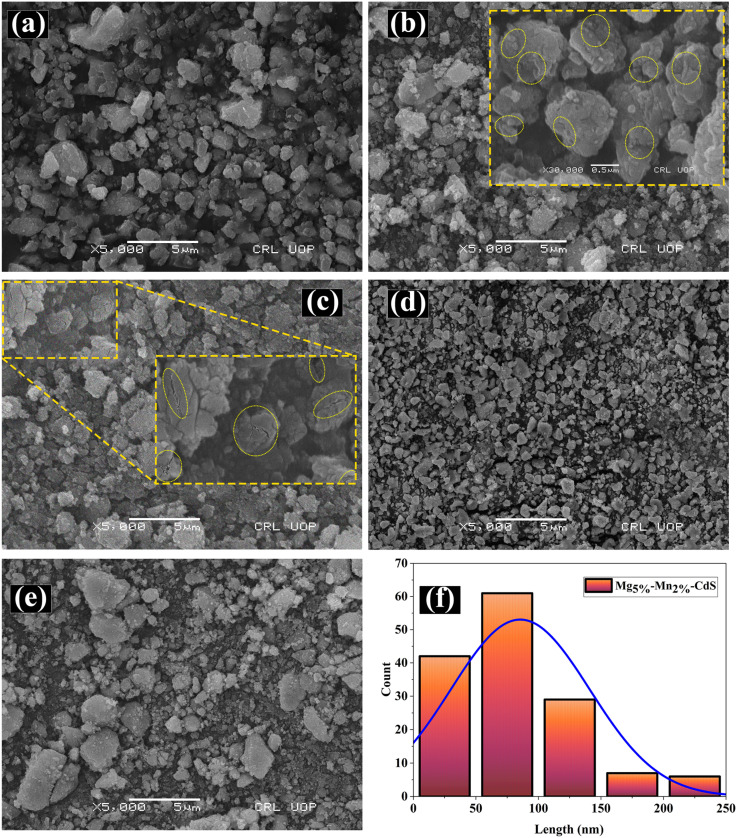
SEM micrographs of (a) pure CdS and (b–e) Mg_*x*_–Mn_2%_-CdS with *x* = 1,3,5, and 7%. (a) The pure CdS sample exhibits agglomerated, irregularly shaped particles. (b and c) Mg incorporation surface cracks and structural defects become evident. (d) At *x* = 5%, the particles are smaller and more uniformly distributed. (e) Further increase to *x* = 7% results in particle coarsening. (f) Particle size distribution histogram of Mg_5%_–Mn_2%_-CdS.

The scanning electron microscopy (SEM) images depict the structural and morphological properties of both pure CdS and Mg–Mn-co-doped CdS, with varying concentrations of Mg at 1%, 3%, 5%, and 7%, while the concentration of Mn is fixed at 2%. Surface morphology and particle size significantly influence photocatalytic performance.^30^ In Fig. 3(a–e), the SEM images are shown.

Fig. 3(a) depicts the surface morphology and particle size of pure CdS. The SEM images suggest nanoparticles with rough surfaces, densely packed and irregularly shaped grains, and a broad size distribution of grains. The poor morphological properties of the grains predict low crystallinity and poor control over particle growth, thus resulting in reduced surface area, fewer active sites, insufficient light harvesting, and less efficient charge carrier separations, which collectively lead to reduced photocatalytic activity.^31^

Fig. 3(b) shows the image obtained from SEM for the Mg_1%_–Mn_2%_-CdS. The SEM analysis depicts variations in the morphology of nanoparticles as a result of co-doping. The grown nanoparticles appear better defined, with microcracks and voids (highlighted in yellow), and the grain size increased significantly. These structural and morphological changes are more likely produced due to the substitution of smaller Mg^2+^ (0.72 Å) and Mn^2+^ (0.67 Å) ions at Cd^2+^ (0.95 Å) lattice sites. This resulted in enhanced active sites and improved light harvesting, thus improving photocatalytic activity.

Fig. 3(c) shows the SEM images of CdS co-doped with 3% Mg co-doping and a fixed 2% Mn concentration. Compared to the Mg_1%_ co-doping, there is a noticeable increase in the growth of cracks and voids. However, the particle size of Mg_3%_–Mn_2%_-co-doped CdS is almost the same as Mg_1%_–Mn_2%_-co-doped CdS. The SEM images reveal surface morphology and particle aggregation, where properties such as agglomeration and micro-irregularity are attributable to sample preparation and growth behavior rather than crystallographic lattice distortion, as quantified by XRD. This enhance charge carrier separation by introducing shallow traps, consequently improving light harvesting and increasing photocatalytic performance.

Fig. 3(d) shows the SEM image obtained for the co-dopant concentration of Mg increased by 5%. The particle size is significantly reduced, and a uniformity is observed throughout, exhibiting enhanced dispersion. The 5% Mg concentration promotes the growth of large CdS particles into smaller, more uniform ones. The 5% concentration of Mg prompts the controlled growth of nanoparticles by introducing strain fields that limit uncontrolled expansion of the grains. The SEM image depicts nanoparticles with smoother surfaces and minimal agglomeration, making the sample optimal for photocatalytic applications due to its enhanced structural and morphological properties.

Fig. 3(e) shows the SEM image of the 7% Mg co-dopant sample. The images indicate a deterioration in morphology; surface roughness increases with shape irregularity, and particles aggregate. This structural and morphological degradation is likely due to oversaturation, resulting in excess lattice strain, phase segregation or secondary phase formation. This causes increased charge carrier recombination and generates a reduced number of active sites, consequently lowering photocatalytic performance. On this basis, there is no direct correlation between the SEM-observed surface features and lattice distortion.

Fig. 3(f) shows a histogram of particle length against their count for the optimal concentration of 5% Mg. The particle length of 73 nm is the center of the Gaussian distribution, conforming to the regular particle size. This shows the increased specific surface area, improved light absorption and reduced diffusion distance of charge carriers, resulting in overall enhanced photocatalytic activity.

### 3.3 EDX (energy-dispersive X-ray)

**Fig. 4:**
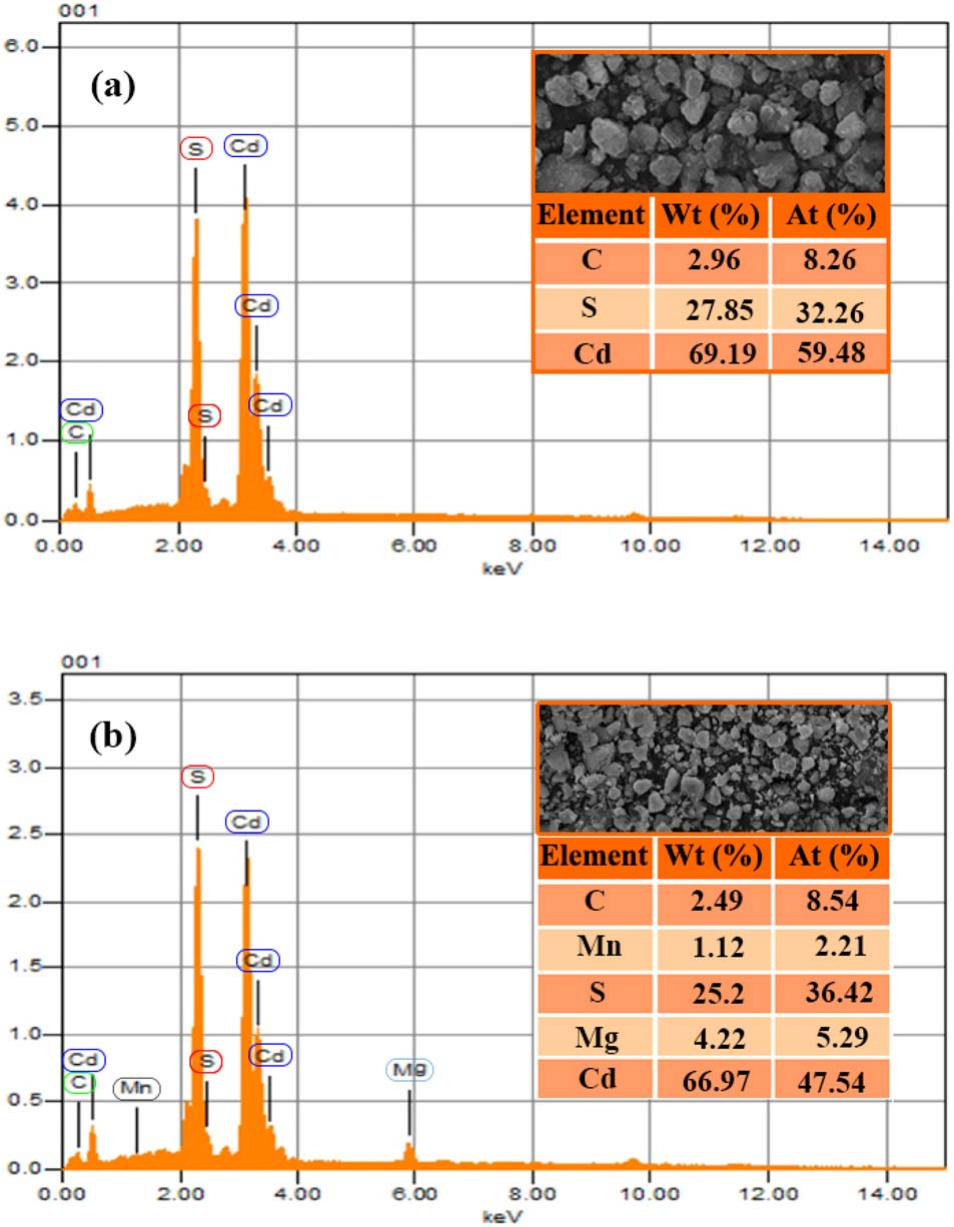
EDX spectra of (a) pure CdS and (b) Mg_5%_–Mn_2%_-CdS showing the elemental composition of the synthesized samples. Insets showing the corresponding SEM micrographs and EDX quantitative analysis (wt% and at%).

The spectrum obtained from the EDX analysis corresponded to peak values at 3.0 keV and 2.3 keV for cadmium (Cd) and sulphur (S), respectively, for pure CdS in Fig. 4(a). An additional carbon (C) peak at 0.27 keV was also observed, most likely resulting from the carbon tape during electron microscopy; this peak does not reflect the actual sample.^32^

For the Mg_5%_–Mn_2%_-CdS sample, similar peaks are observed in Fig. 4(b) for cadmium (Cd) and sulphur (S) in addition to magnesium (Mg) at 1.3 keV and manganese (Mn) at 5.9 keV, which confirmed successful co-doping of Mg and Mn into the CdS matrix. Again, the carbon peak is observed due to the carbon tape used in CdS. These findings highlight the importance of careful sample preparation and sample handling while interpreting EDX data to avoid contamination and oxidation, ensuring accurate determination of elemental composition.

Although EDX is used to establish the presence and homogeneity of Mg and Mn within the CdS matrix, X-ray photoelectron spectroscopy (XPS) is further used to determine the presence and oxidation states of the dopants on the surface by analyzing the corresponding core-level signals. The analysis using EDX reveals the existence and the relative abundance of Mg and Mn dopants.

### 3.4 XPS analysis

**Fig. 5:**
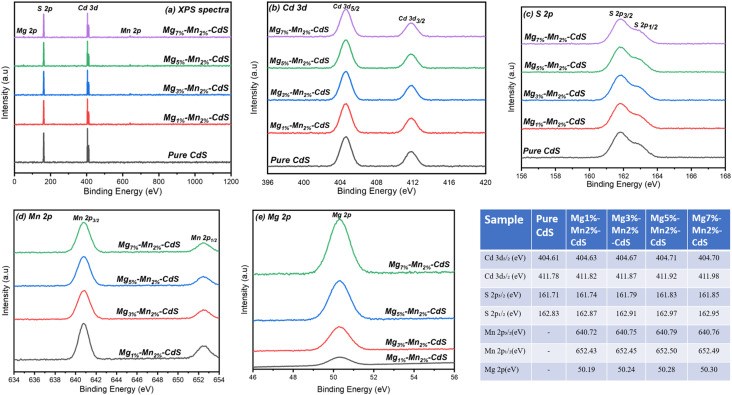
XPS spectra of pure CdS and Mg–Mn co-doped CdS samples with (a) the survey scan spectra, (b) high-resolution deconvoluted spectra of Cd 3d, (c) S 2p, (d) Mn 2p, and (e) Mg 2p. The binding energies are summarized in the table. The positive shift in the Cd 3d and S 2p peaks with increasing Mg content confirms the lattice incorporation of dopants and charge redistribution between Cd–S and Mg/Mn–S sites.

XPS analysis was utilized for the investigation of the modifications caused by co-doping of Mg and Mn in CdS nanoparticles in the surface composition, oxidation states, and electronic structure. Fig. 5 shows a comparison of XPS spectra of the pure and co-doped samples of CdS. The co-doped ratios (Mg_1%_–Mn_2%_-CdS, Mg_3%_–Mn_2%_-CdS, Mg_5%_–Mn_2%_-CdS, and Mg_7%_–Mn_2%_-CdS) show binding energies ranging from 0 to 1200 eV (Fig. 5(a)). The Cd, S, Mg and Mn peaks being prominent in the spectrum ensures the purity of the sample and absence of impurities like carbon and oxygen.

In the spectrum of CdS (Fig. 5(b)), two well-defined peaks appear at energies 404.61 eV and 411.78 eV, corresponding to the spin–orbit components of Cd 3d_5/2_ and Cd 3d_3/2_, respectively. This indicates the divalent oxidation states of Cd^2+^ in the pure CdS. The doping results in a slight shift in these energies to 404.68 eV and 411.98 eV for the Cd 3d components in dopant ratios (up to 404.68 eV and 411.98 eV for Mg_7%_–Mn_2%_-CdS) as a result of changes induced in the electronic properties by co-doping. The Mg^2+^ and Mn^2+^ being less electronegative than Cd^2+^ results in a positive shift and partial redistribution of charges along with a slight increase in the ionic character of the Cd–S bond.

The peaks at 161.71 and 162.83 eV in the S 2p spectra of pure CdS (Fig. 5(c)) correspond to the orbits S 2p_3/2_ and S 2p_1/2_, respectively, characteristic of sulphide ions (S^2−^) bound to Cd^2+^. Co-doping resulted in a shift in energies to 161.88 eV and 162.95 eV for Mg_7%_–Mn_2%_-CdS, as a result of perturbations in the S–Cd bond caused by the incorporation of Mg and Mn into lattice sites. There were no peaks at binding energies of 168–170 eV, which are associated with sulphate or other oxidized sulphur species, and this suggests that the sulphide anion was stable under the conditions of the synthesis process. On the other hand, the doped samples of CdS reveal peaks at 640.72 eV and 652.43 eV (Fig. 5(d)), characteristics of Mn 2p_3/2_ and Mn 2p_1/2_, respectively, and the spin–orbit separation is ∼11.7 eV, which corresponds to the Mn^2+^ oxidation state. No other peaks of higher binding energies are observed, meaning the absence of higher oxidation states and Mn^2+^ are replaced with Cd^2+^ in the CdS lattice. Mg 2p at 50.3 eV corresponds to the Mg^2+^ oxidation state (Fig. 5(e)), which favors the sulphide environment; increasing the concentration of Mg leads to an increase in Mg 2p peak intensity, confirming the successful incorporation of Mg into the CdS lattice. The uniformity of Mg and the absence of oxide or hydroxide peaks confirms successful doping and minimal surface oxidation. Overall, the XPS analysis confirms the successful incorporation of Mg^2+^ and Mn^2+^ in the CdS lattice and the absence of any other oxide impurities, preserving the stoichiometric phase of CdS.

The positive phase shift in binding energies of Cd 3d and S 2p with higher Mg concentrations confirms the modification of the band structure, meaning enhancement in electron density and strain effects in the lattice. These changes are consistent with observed bandgap modifications and improved charge separations observed in optical and photocatalytic studies. As a result, this analysis is evidence of the chemical incorporation and electronic interactions of the dopant ions with the host lattice, playing a crucial role in photocatalytic and antimicrobial activities.

### 3.5 Photoluminescence spectroscopy (PL)

**Fig. 6:**
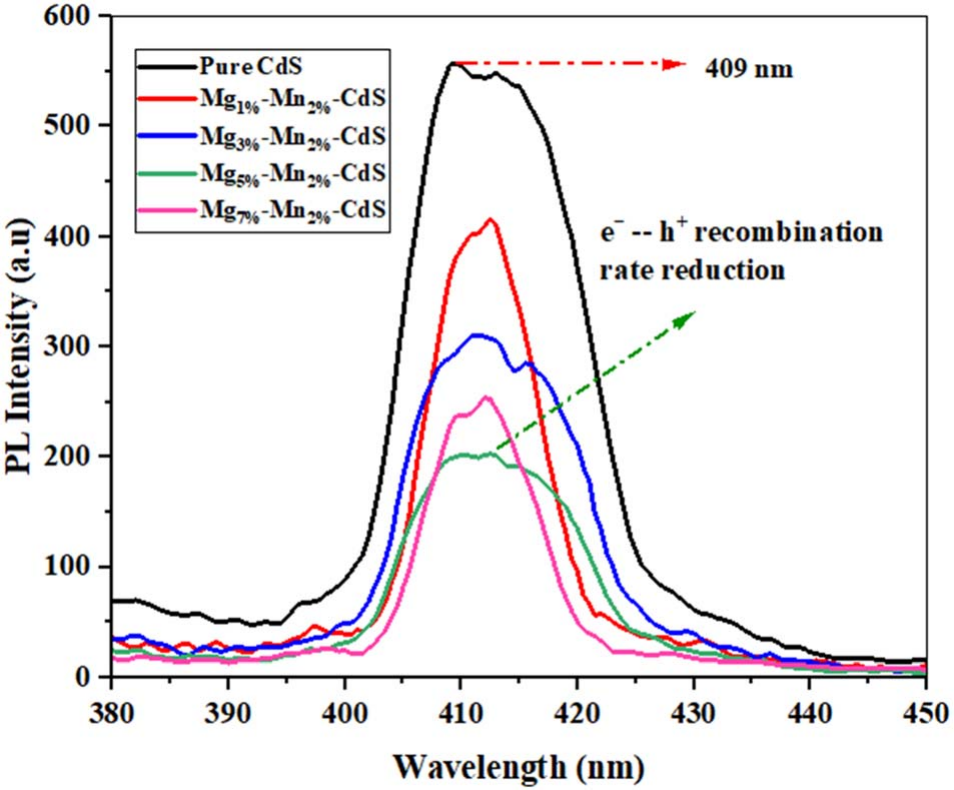
PL analysis spectra of pure CdS, Mg_1%_–Mn_2%_-CdS, Mg_3%_–Mn_2%_-CdS, Mg_5%_–Mn_2%_-CdS and Mg_7%_–Mn_2%_-CdS nanomaterials.

Photoluminescence (PL) spectroscopy was carried out to study the effects produced by Mg and Mn co-doping on the recombination rates of electron–hole (e^−^–h^+^) pairs. Fig. 6 shows the peak obtained for the pure CdS at ∼410 nm, indicating high PL intensity and significant recombination rates for e^−^–h^+^ pairs. The Mg and Mn–co-doped samples of CdS showed a decrease in the PL intensity, indicating reduced e^−^–h^+^ recombination rates.^33^ In the Mg_1%_–Mn_2%_-CdS sample, a slight decrease in peak intensity was observed compared to pure CdS, and the reduction became more pronounced with increasing doping concentrations of Mg. The Mg_5%_–Mn_2%_-CdS sample exhibited the lowest PL intensity, corresponding to a substantial reduction in the recombination rates of electron–hole pairs, and consequently, improving photocatalytic activity.

However, further increasing the doping concentrations of Mg to 7% (*e.g.*, Mg_7%_–Mn_2%_-CdS) led to high PL peak intensity, marking increased recombination rates. This may result from the new defect sites introduced by increasing co-dopant concentrations or crystallographic imperfections, leading to enhanced electron–hole recombination.^34^ Therefore, we can say that further increasing the concentration of Mg beyond 5% while keeping Mn at 2% can reverse the beneficial effects. The photocatalytic properties for the 7% doping concentration were poor compared to 5%.

For the optimal doping concentration, Mg_5%_–Mn_2%_-CdS, the reduced PL intensity indicates that Mg and Mn introduced new defect states that could trap electrons, thus enhancing electron–hole pair separation and reducing recombination rates, consequently increasing photocatalytic performance significantly.^35^ In contrast, the increase in PL intensity at higher concentrations of the co-dopant (*e.g.*, Mg_7%_–Mn_2%_-CdS) introduced defects that supported recombination, thus resulting in reduced photocatalytic properties.^36^ These outcomes are consistent with the literature, highlighting the role of doping concentrations in recombination rates in semiconducting materials.

The decreased PL intensity in the co-doped CdS samples indicates inhibition of radiative recombination of photogenerated charge carriers. It is important to note that steady-state PL measurements are mostly used to measure recombination-related behavior of emission and not to directly measure charge transport or separation efficiency. Therefore, PL quenching is in this case interpreted as an indicative trend of decreased electron–hole recombination instead of conclusive evidence of increased charge separation. It is admitted that steady-state PL spectroscopy does not allow for the conclusive determination of the separation or transport dynamics of charge carriers. Advanced techniques, *e.g.*, electrochemical impedance spectroscopy (EIS), transient photocurrent response or time-resolved photoluminescence, would provide more direct insights into interfacial charge transfer processes. These analyses are beyond the scope of the present study and will be conducted in future investigations.

### 3.6 FTIR studies

**Fig. 7:**
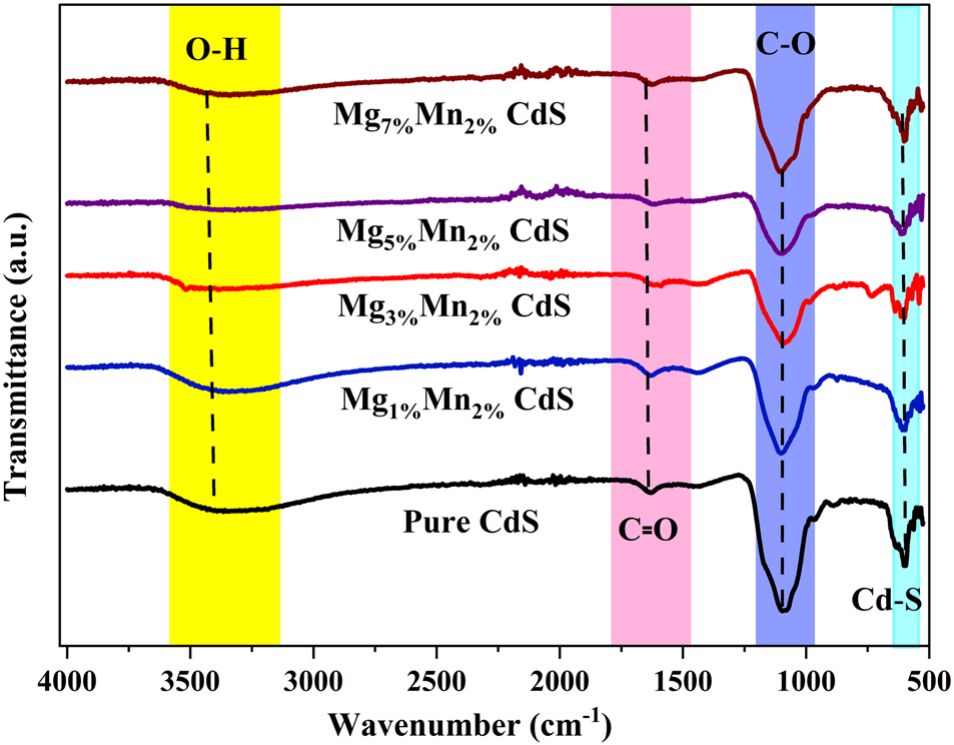
FTIR spectra of pure CdS and Mg–Mn co-doped CdS with different doping ratios.

FTIR spectroscopy was employed to investigate the vibrational modes and surface functional groups of pure CdS and Mg–Mn co-doped CdS nanostructures. Fig. 7 shows the FTIR spectra in the range of 4000–500 cm^−1^. The prominent absorption band around 610–615 cm^−1^, present in all samples, corresponds to the Cd–S stretching vibration, confirming the formation of the CdS lattice structure.^37,38^ Small peak shifts observed in the doped samples indicate changes in particle size and lattice strain due to the presence of larger Mg^2+^ and Mn^2+^ ions. A broad band near 3400 cm^−1^, attributed to O–H stretching vibrations, indicates the presence of surface-bound hydroxyl groups or adsorbed moisture.^39,40^ This band weakens with increasing Mg content, suggesting reduced surface hydroxylation and improved crystallinity. Weak absorptions between 2800 and 2950 cm^−1^ are linked to C–H stretching vibrations, possibly from residual organic species or surfactants used during synthesis.^41^ These peaks diminish in intensity in the doped samples, implying improved surface purity. Medium-intensity bands around 1550–1650 cm^−1^ are assigned to H–O–H bending vibrations of molecular water, which also decrease with higher dopant concentrations, indicating reduced surface-bound water.^42^ In the pure CdS sample, strong peaks near 1639 cm^−1^ (C=O) and 1125 cm^−1^ (C–O) appear, likely due to surface oxidation or contamination.^37,43^ The significant reduction or disappearance of these bands in co-doped samples indicates excellent surface passivation and the successful elimination of organic substances. These spectral changes, measured under the same experimental conditions, verify the successful co-doping of Mg and Mn into the CdS host material.

The FTIR spectra provide data about the chemical surface environment and the bonding features of the synthesized samples. The measured vibrational characteristics and slight shifts in the bands with the addition of Mg/Mn prove alterations in interactions on the surface and local bonding environments. It must however be noted that FTIR spectroscopy does not directly verify the substitutional incorporation of dopant ions into the CdS lattice. The structural incorporation of Mg^2+^ and Mn^2+^ is more reliably established by systematic shifts in XRD peaks and the appearance of typical core-level signals in the XPS analysis.

### 3.7 UV-visible spectrum

**Fig. 8:**
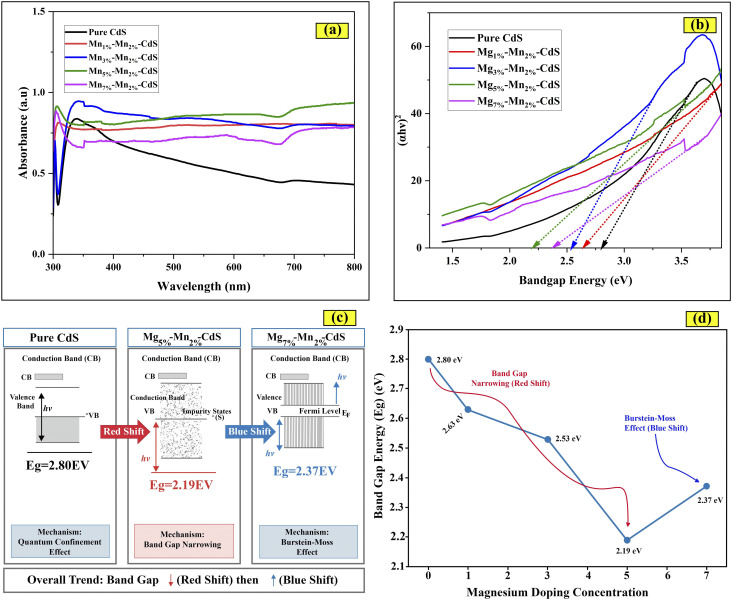
(a) UV-vis spectra of pure CdS, Mg_1%_–Mn_2%_-CdS, Mg_3%_–Mn_2%_-CdS, Mg_5%_–Mn_2%_-CdS and Mg_7%_–Mn_2%_-CdS. (b) Tauc plot used to determine the energy gap of the synthesized materials. (d) Effect of Mg concentration on the energy band gap. (c) Band gap narrowing and widening due to the Burstein–Moss effect.

The band gap energies of pure CdS and Mn–Mg-co-doped CdS were characterized by ultraviolet-visible spectroscopy. This technique measures the interaction of light within the wavelength range of 300 to 800 nm, as shown in Fig. 8(a). The absorption peaks at 442, 469, 490, 568 and 523 nm correspond to electron transitions from the valence band to the conduction band in the nanostructures at the specified wavelengths. According to the Tauc plots in Fig. 8(b), the band gap energy of pure CdS was found to be 2.8 eV, whereas the band gaps for Mg_1%_–Mn_2%_-CdS, Mg_3%_–Mn_2%_-CdS, Mg_5%_–Mn_2%_-CdS and Mg_7%_–Mn_2%_-CdS were 2.63 eV, 2.53 eV, 2.19 eV and 2.37 eV, respectively. As the concentration of doped magnesium and manganese increased, Mg_1%_–Mn_2%_-CdS, Mg_3%_–Mn_2%_-CdS, Mg_5%_–Mn_2%_-CdS and Mg_7%_–Mn_2%_-CdS show less bandgap energy compared to pure CdS. The decrease in the bandgap of the Mg–Mn-co-doped CdS indicates the generation of extra energy states below the conduction band and above the valence band.^44^ The bandgap interestingly increased from 2.19 eV to 2.37 eV when the Mg concentration was raised to 7% (Fig. 8(d)). This is due to the Burstein-Moss effect, in which the Fermi energy level moves toward the valence band with increasing concentration of impurity atoms in the semiconductor and causes a blue shift (Fig. 8(c))).^45^ The observed photocatalytic activity can be attributed to the improved absorption of light in the visible range due to the narrowed band gap of co-doped samples.

The optical band gap (*E*_g_) of the synthesized samples was determined using the Tauc relation as follows:(*αhν*)^*n*^ = *A*(*hν* − *E*_g_),where *α* is the absorption coefficient, *hν* is the photon energy, *A* is a proportionality constant and *n* = 2 for direct allowed electronic transitions. CdS is a well-established direct bandgap semiconductor, so plots of (*αhν*)^2^*versus hν* were used. The linear portion near the absorption edge was carefully chosen and extrapolated to intersect the energy axis ((*αhν*)^2^ = 0), and the intercept was considered the optical bandgap. The fitting range and intercepts are now explicitly marked in the revised Tauc plots in order to prevent ambiguity.

The effects of quantum confinement associated with the nano-sized particles are the reason for the observed increase in the band gap of pure CdS (2.80 eV) compared to the bulk CdS (2.42 eV). This approach follows standard practice for direct bandgap semiconductors and has been widely adopted in recent studies of CdS-based photocatalysts.

## 4. Photocatalytic activity

The dye degradation and efficient performance of the synthesized samples were assessed utilizing a custom-built photocatalytic reactor that uses a 300 W Xenon (Xe) arc lamp and a visible light cutoff filter (*λ* > 420 nm) at the Nanotechnology Lab, Physics Department, the University of Gujrat, Pakistan. A calibrated radiometer with a sensor was placed at the sample position to measure irradiance, and the resulting value was 250 W m^−2^. The vertical distance of the lamp from the sample was 16 cm, which was at a distance deemed acceptable for the range of 12–20 cm. The reactor was cylindrical (7 cm diameter × 20 cm height), which ensured uniform exposure to the light.

The catalytic efficiency primarily relies on the time of contact with the photocatalyst. However, performance is also affected by several other key factors, including recombination of electron–hole pairs, particle size and morphology, pollutant concentration, temperature, and band gap.^46–48^

**Fig. 9:**
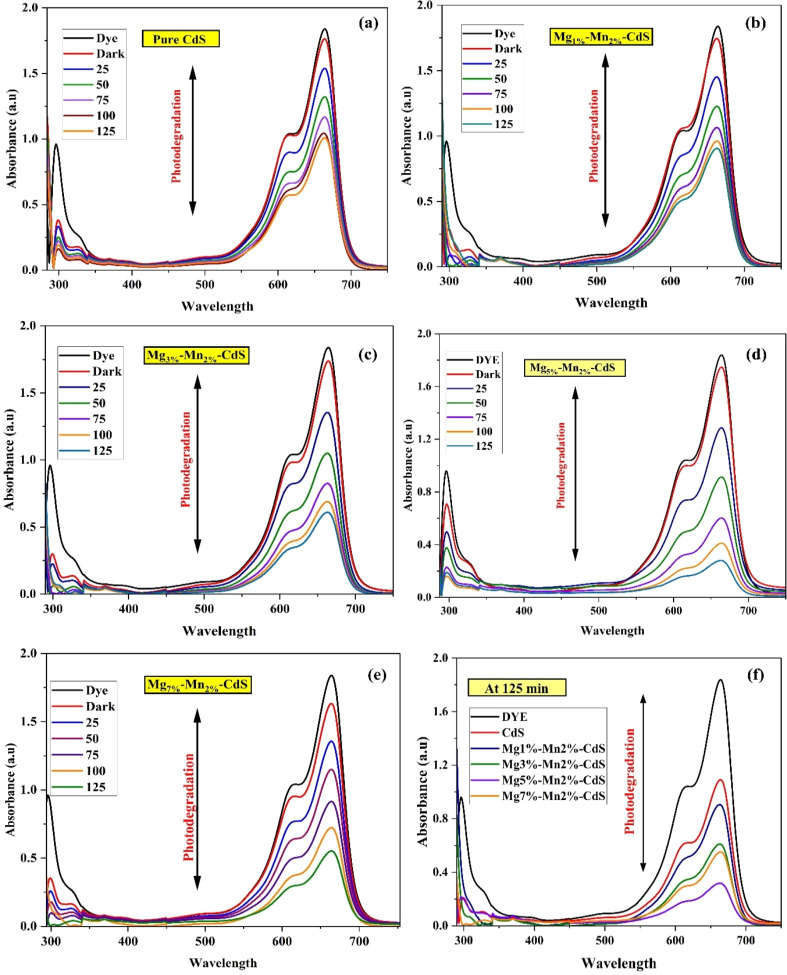
(a–e) Absorbance spectra of pure CdS and Mg_1%_–Mn_2%_-CdS, Mg_3%_–Mn_2%_-CdS, Mg_5%_–Mn_2%_-CdS and Mg_7%_–Mn_2%_-CdS, respectively. The degradation after 125 minutes for all compounds is shown in (f). An initial 25 minutes adsorption–desorption period was allowed to achieve stabilization.

A solution of dye with a concentration of 10 ppm was prepared by dissolving it in distilled water. Each 100 mL dye solution sample containing 0.1 mg of photocatalyst was stirred continuously in the absence of light for 25 minutes to gain adsorption equilibrium. The reaction solution was placed in the reactor, subsequently positioned at a distance of 16 cm from the UV-visible light source. Absorption spectra were recorded at 25 minutes intervals to monitor the decolonization of the solution, which indicates photocatalytic activity. A 5 mL sample of the solution was extracted at 25 minutes intervals and analyzed using a UV-visible double-beam spectrophotometer. This process was reiterated over a total duration of 125 minutes. The degradation efficiency of the dye was calculated using the following equations:

2 Degradation (%) = 1 − (*C*/*C*_o_) × 100,

3 Rate constant (*k*) = ln(*C*_o_/*C*),

where *C*_o_ denotes the concentration at the initial time, while *C* represents the dye concentration measured at a specific time *t*. Fig. 9 shows the UV-visible spectra of pure and Mg–Mn-co-doped CdS in a methylene blue (MB) solution after 125 minutes. The continuous decline in peak absorption over time clearly indicates the gradual degradation of MB.

As shown in Fig. 9, during the degradation process, Mg_5%_–Mn_2%_-co-doped CdS consistently enhances degradation efficiency. Therefore, it is noteworthy that the photocatalytic degradation performance of Mg_5%_–Mn_2%_-co-doped CdS improved over time and eventually extended into the visible light region. This improvement results from a significant reduction in the band gap energy of the synthesized nanoparticles, leading to modifications in their electronic structure. The present study investigated Mg_*x*_-Mn_2%_-co-doped CdS nanoparticles with Mg concentrations of 1%, 3%, 5%, and 7%. The nanoparticles degraded methylene blue by 42%, 54.66%, 68%, 84.7%, and 77%, respectively, as shown in Fig. 9(a–f), demonstrating a clear improvement in photocatalytic activity compared to pure CdS. Mg_5%_–Mn_2%_-co-doped CdS achieved the highest degradation of methylene blue dye within 125 minutes. Photocatalytic degradation efficiencies, kinetic rate constants, and regression coefficients (*R*^2^) for all samples are provided in Table S2 (SI).

The complementary effects of Mg^2+^ and Mn^2+^ co-doping are further supported by the structural and optical observations.

The lattice contraction observed from XRD peak shift can be mainly explained by the substitution of Mg^2+^, and the gradual red-shift in optical absorption and PL quenching due to Mn incorporation can be explained by alterations in electronic states and recombination mechanisms. It is interesting to note that co-doped samples are characterized by a balanced structural distortion and optical response, which implies that the effect of Mg^2+^ modulation on faults and the effect of Mn^2+^ on electron work together. This synergistic interaction is the reason why co-doped system performs optimally without necessarily involving excessive recombination caused by defects.

Even though several reported photocatalysts attain faster rates of degradation when operated under optimized laboratory conditions, the systems tend to utilise high catalyst concentrations, UV irradiation, or intricate multi-constituent structures. In the present work, the Mg–Mn-co-doped CdS system is proposed as a multi-functional platform that can simultaneously show photocatalytic activity under visible light and antibacterial performance, achieved by a simple synthesis route and low dosage of catalysts. When performance is evaluated in the context of catalyst loading, irradiation source, and overall material functionality, an overall favorable balance on the performance aspects of efficiency, simplicity, and practical applicability for wastewater treatment is demonstrated by the present system.

**Fig. 10:**
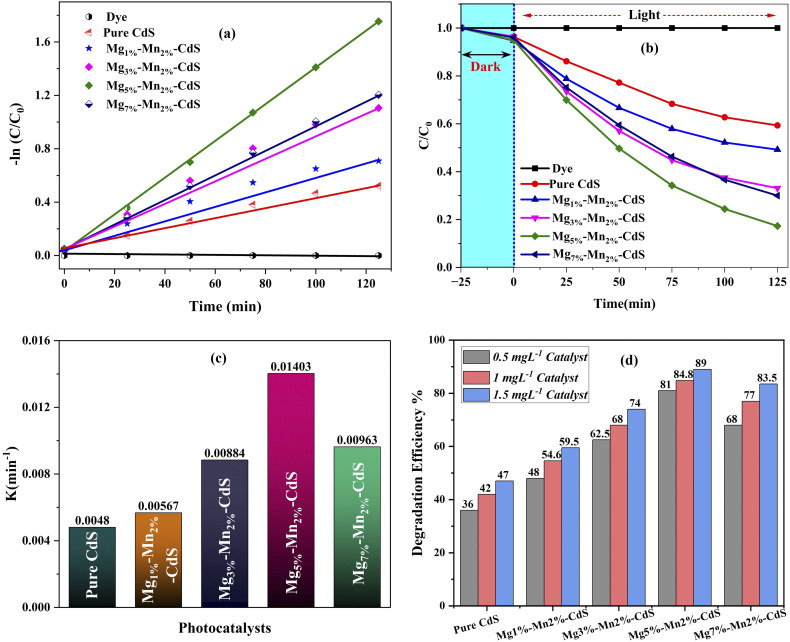
Photocatalytic performance of pure CdS and Mg–Mn-co-doped CdS: (a) kinetic plots for dye degradation, (b) photodegradation curves, (c) rate constants (*k*) of photocatalysts and (d) effects of catalyst concentration on the efficiency of dye degradation.

The concentration of Mg doping plays a crucial role in the photocatalytic activity of Mn_2%_-doped CdS. At low concentrations (1% and 3%), the content of Mg is too low to introduce effective surface defects or modify the electronic structure, allowing minimal charge separation and reduced degradation efficiency. Higher doping (7%) leads to excess deformation of the lattice, agglomeration of the particles and formation of recombination centers, which reduce photocatalytic performance. The Mg_5%_–Mn_2%_-co-doped CdS sample achieves the best compromise and improves visible-light absorption, surface reactivity and charge carrier separation, therefore showing the highest degradation efficiency (Fig. 10(d)). The slightly reduced photocatalytic activity (85%) of Mg_5%_–Mn_2%_-co-doped CdS with respect to the reported literature, 98% for Mg-doped CdS, 88% for Mn-doped CdS in photodegradation of methylene blue,^24,49^ can be explained by variations in synthesis conditions, doping efficiency, surface area, and experimental setup (*e.g.*, light source and dye concentration). Additionally, crystallinity, morphology, and charge separation capability also play significant roles in determining degradation efficiency. SEM analysis of Mg_5%_–Mn_2%_-co-doped CdS nanoparticles shows a partially fractured and porous crystal structure, which increases surface roughness. The structural defects and pores improve photocatalytic performance by offering a larger surface area and more active sites, enhancing dye adsorption and efficient separation of photo-generated charge carriers. Nonetheless, over-agglomeration is prevented to maintain an active morphology appropriate for high photocatalytic degradation efficiency.

Applying first-order kinetics, calculations of the rate constant using eqn (2) and evaluations of the correlation coefficient (*R*^2^) were conducted (Fig. 10(a)). The highest *R*^2^ value was observed for Mg_5%_–Mn_2%_-co-doped CdS since this composition is the most effective charge separator and has a stable surface, which results in the photocatalytic degradation closely following first-order kinetics. Other ratios were not able to strike this balance, resulting in deviations from ideal kinetics and lower *R*^2^ values. The rate constant values against the photocatalysis of all these samples shown in Fig. 10(c). Linear fitting performed in Origin Pro 2025 are listed in Table S3.

In order to ensure that the observed degradation of methylene blue (MB) was due purely to photocatalytic action and not simply adsorption or a direct photolysis reaction, two control experiments were carried out under the same experimental conditions. The experiments were conducted in the form of dark adsorption (*i.e.*, stirring with no light irradiation) and photolysis (*i.e.*, exposure of the MB solution to visible light without the presence of a catalyst). Photocatalytic and control experiments were also conducted in triplicate (*n* = 3) to confirm reliability, and the findings are reported in the form of the mean ± standard deviation (SD).

**Fig. 11:**
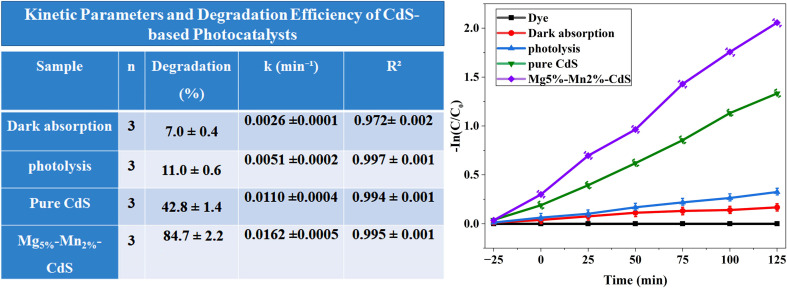
Kinetically validated photolysis and dark adsorption controls of CdS-based photocatalysts.

The efficiency of degradation was calculated *via*eqn (2) whereas the kinetic behavior was determined *via* the pseudo-first-order model explained earlier in eqn (3). The respective kinetic plots of ln(*C*_0_/*C*) as a function of irradiation time (*t*) are shown in Fig. 11, and the rate constants (*k*) and correlation coefficients (*R*^2^) are summarized in Table S2. All samples exhibited high linearity, with *R*^2^ values ranging from 0.97 to 0.99, confirming that the photodegradation process follows pseudo-first-order kinetics.

The control experiments showed negligible degradation (less than 10% in 125 minutes), which means that there was no direct photolysis and no remarkable absorption at these conditions. In contrast, the photocatalyst samples showed significant degradation efficiencies. Pure CdS achieved a maximum degradation of 42% ± 1.4% with a rate constant (*k*) of 0.0110 ± 0.0004 min^−1^ and a correlation coefficient *R*^2^ of 0.994. Conversely, the Mg_5%_–Mn_2%_-CdS sample demonstrated the greatest photocatalytic degradation rate of 84.7% ± 2.2% with *k* = 0.0162 ± 0.0005 min^−1^ and *R*^2^ = 0.995 under visible-light irradiation for 125 min.

The large correlation coefficients confirm that the reaction occurs mainly through a single kinetic pathway that is specified by the dynamics of photoinduced charge carriers. The observed larger rate constant and increased degradation efficiency of Mg_5%_–Mn_2%_-CdS indicate that Mg and Mn–co-doped CdS exhibit a synergizing activity, enhancing visible-light absorption, charge separation of charges, and the production rate of reactive oxygen species (˙O_2_^−^ and ˙OH). The kinetic analysis shown in Table S2 and Fig. 10(a and b) is remarkable and reproducible because it includes photolysis and dark adsorption controls and is supported by triplicate statistical validation (mean ± SD).

The linear plots of ln(*C*_o_/*C*) *vs.* time and summarized kinetic parameters (*k*, *R*^2^, and degradation percentage) reveal that there is no appreciable degradation (<10%) under the control conditions, which proves that MB removal is a result of photocatalytic activity. It is accepted that UV-vis spectroscopic monitoring of dye decolourization is indicative of mineralization but not totally conclusive of complete mineralization. Quantitative evaluation of mineralization using the total organic carbon (TOC) analysis will be the topic of future investigation. Based on the obtained photocatalytic behaviour and previously reported CdS-based photocatalysts, a possible degradation route for methylene blue is proposed. Under the action of visible light, photoexcited electrons and holes produce reactive oxygen species (such as superoxide radicals (˙O_2_^−^) and hydroxyl radicals (˙OH), which lead to the initiation of the oxidation cleavage of the chromophoric structure of methylene blue. This process results in the formation of intermediate species by stepwise demethylation, ring opening and fragmentation reactions, culminating in smaller organic molecules. While it is not possible to directly identify the intermediates in the present study, the proposed pathway is in line with the literature reports on dye degradation in the semiconductor regime.

### 4.1 Recycling stability and efficiency

**Fig. 12:**
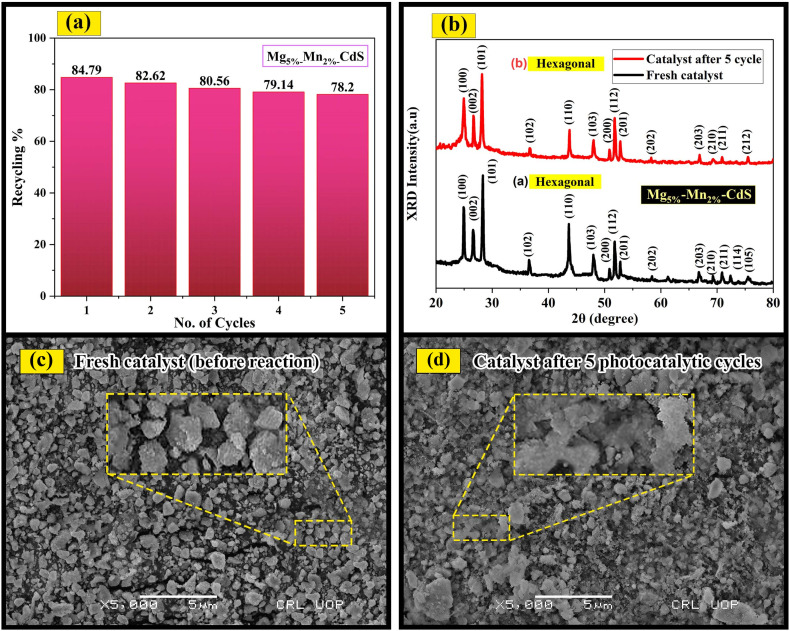
(a) Reusability of Mg_5%_–Mn_2%_-CdS over five cycles for the photocatalytic degradation of Mb dye. (b) XRD patterns for fresh and reused catalysts, revealing stability of the hexagonal structure. (c) SEM image of the catalyst prior to any reaction (fresh catalyst). (d) SEM image of the catalyst after five cycles, showing slight agglomeration compared with the original morphology.

A number of experiments were carried out to investigate the response stability and recyclability of Mg_5%_–Mn_2%_-co-doped CdS over repeated uses. After each photodegradation cycle, the recovered catalyst was washed with ethanol and deionized water, then dried at 80 °C for three hours before reuse. The photocatalyst retained high stability with minimal drop in photocatalytic efficiency over five cycles, as shown in Fig. 12(a). The removal efficiencies were 84.79%, 82.62%, 80.56%, 79.14%, and 78.2% for cycles 1 to 5, respectively. The loss in efficiency is minor and due to the deposition of intermediate and detrimental surface species on the catalyst, which partially block the active sites.

XRD analysis was used to further evaluate the phase stability of the recycled catalysts (Fig. 12(b)). Both fresh and reused catalysts exhibited sharp diffraction peaks associated with the hexagonal structure of CdS and do not show evidence of any new impurity phases. This indicates that the crystal structure remained unchanged even after five photocatalytic cycles. Moreover, comparison of the diffraction intensities indicates that Mg_5%_ and Mn_2%_ co-doping exerted a stabilizing effect on the CdS lattice in terms of structural changes during the recycling process.

Surface morphology analysis of Mg_5%_–Mn_2%_-co-doped CdS using SEM is shown in Fig. 12(c and d). The fresh catalyst exhibited nanoparticles with irregular but well-defined boundaries, displaying good dispersion and uniform particle formation. After five photocatalytic cycles, only slight agglomeration and surface roughening could be observed, most probably due to light-induced reactions and adsorption of residual by-products on the catalyst surface during the repeated cycles. Still, the particle structure was stable, and no noticeable fusion or deformation was detected, which means that the catalyst retained its morphological integrity and durability even after repeated use.

In general, the Mg_5%_–Mn_2%_-co-doped CdS photocatalyst revealed excellent recyclability and durability over time, with almost no weight loss, showing that it could be used in real wastewater treatment and environmental decontamination applications. Surface activity could be further maintained by regeneration through mild oxidation and long-term thermal treatment.

### 4.2 Photocatalytic mechanism

During photocatalytic activity, when the photocatalyst is irradiated with light exceeding a threshold frequency, electrons in the valence band become excited and transition to the conduction band. This excitation simultaneously creates an equal number of positive charge carriers within the valence band. Photo-generated electrons in CdS react with surface species, resulting in the production of reactive oxygen species (ROS), like superoxide anion radicals, hydrogen peroxide, oxygen ions, and hydroxyl ions,^50^ as shown in Fig. 12. Consequently, CdS effectively inhibits electron–hole recombination by capturing charge carriers, which reduces the overall recombination rate. The reaction formulations are as follows:

4 Mg–Mn-CdS + *hν* → Mg–Mn-CdS (eCB^−^ + *hν*B^+^),

5 Mg–Mn-CdS (*hν*B^+^) + H_2_O → Mg–Mn–CdS + H^+^ + OH^−^

6 Mg–Mn-CdS (eCB^−^) (s) + O_2_ → Mg–Mn-CdS + O_2_˙

7 Mg–Mn-CdS (*hν*B^+^) + OH^−^ → Mg–Mn-CdS + OH˙

8 MB dye + O_2_˙ → degraded products + CO_2_ + H_2_O

9 MB dye + OH˙ → degraded products + CO_2_ + H_2_O

Water is ionized into OH^−^ and H^+^ ions in the presence of the Mg_5%_–Mn_2%_-co-doped CdS catalyst under light irradiation, and the holes are then reacted with water to form positively charged species, which interact with methylene blue dye. When oxygen (O_2_) reacts with electrons, it forms superoxide anion radicals (˙O_2_^−^), and hydroxide ions (OH^−^ ions) react with other radical species to generate peroxide radicals and protons. Subsequently, these reactive intermediates react with superoxide anions and hydroxyl radicals to form potent oxidizing substances. These oxidants then degrade methylene blue (MB) into harmless compounds such as carbon dioxide (CO_2_) and water.^54^Fig. 10 shows the general photocatalytic degradation process. The concentration of methylene blue (MB) decreases over time under light irradiation with Mg_5%_–Mn_2%_-CdS photocatalyst and both in the presence and in the absence of substances that capture radicals, such as ascorbic acid and isopropyl alcohol. The significant reduction in degradation rate caused by these substances confirms that superoxide (˙O_2_^−^) and hydroxyl radicals (˙OH) are the main reactive agents responsible for breaking down MB.

**Fig. 13:**
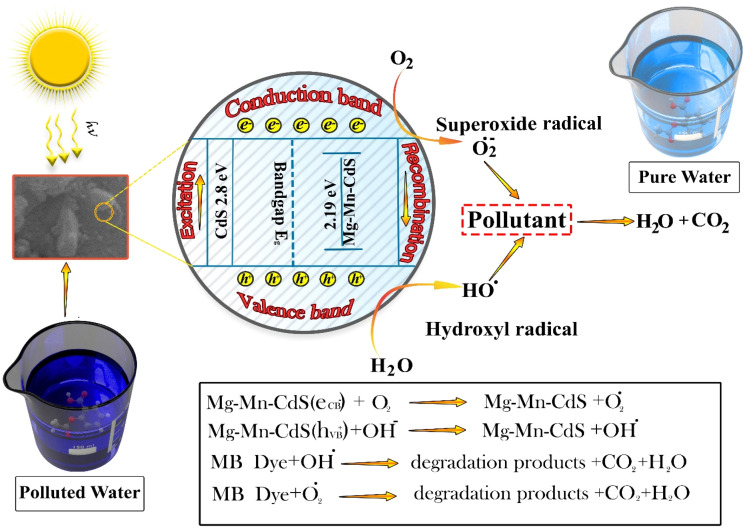
Illustration of the photocatalytic inactivation of pollutants with reactive Mg–Mn-CdS.

The increased efficiency is attributed to lattice strain and active-site engineering resulting from co-doping of Mn and Mg, promoting charge separation and electron transfer during the photocatalytic reaction. Dopant ions and the CdS lattice synergies induce the generation of defect states acting as high-efficiency trapping sites for photogenerated charge carriers, suppressing recombination loss and expanding light absorption into the visible region. The enhancement in redox functionality and the resulting improvement in photocatalytic efficiency due to co-doping arise from the promotion of charge migration across the interface. Similar structural and electron modulations have been documented for analogous nanostructured catalysts prepared by metal–organic frameworks (MOFs), where charge transfer by defects and redox-active surface sites substantially escalates catalytic reactions (Fig. 13).^51^

## 5. Trapping experiment

**Fig. 14:**
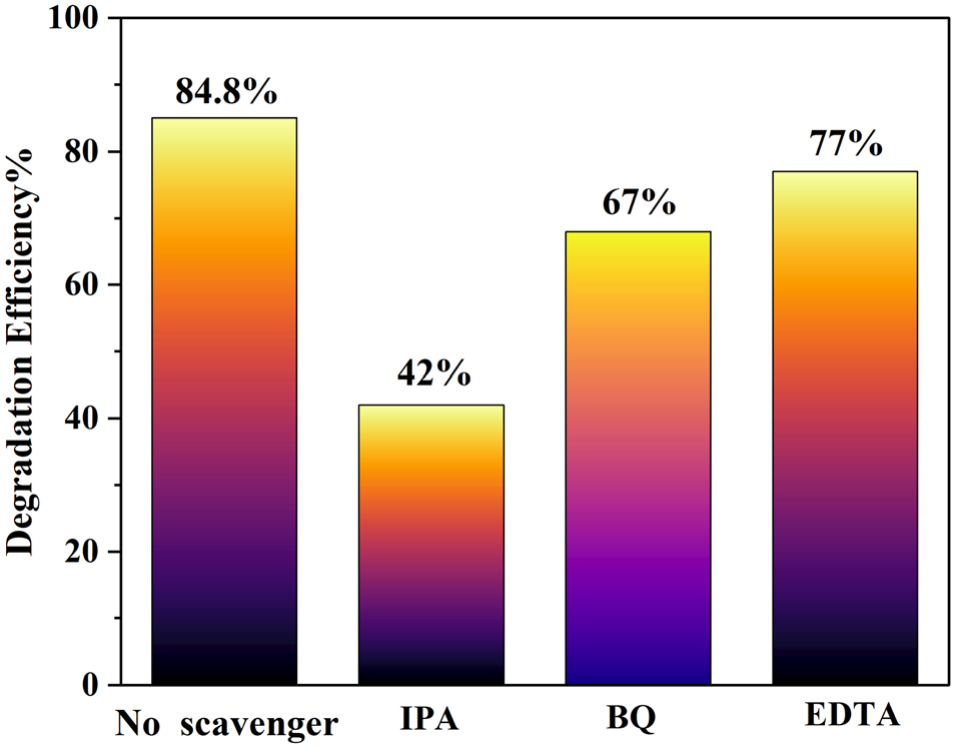
Analysis of the photocatalytic decomposition of Mg–Mn-co-doped CdS, showing the dominant reactive species in MB dye degradation with IPA (˙OH radical scavenger), BQ (˙O_2_^−^ radical scavenger), and EDTA (h^+^ scavenger).

Radical scavenger experiments were performed for the identification of the active species involved in the degradation of MB dye using Mg and Mn-co-doped CdS under visible light (Fig. 14).

Isopropanol (IPA, 0.1 M) was utilized as a hydroxyl radical (˙OH) scavenger, benzoquinone (BQ, 5 mM) as a superoxide radical (˙O_2_^−^) scavenger, and ethylenediaminetetraacetic acid (EDTA, 5 mM) as a hole (h^+^) scavenger. Each scavenger is added separately to the dye solution before light irradiation, while other parameters were kept identical to the control experiment (without scavenger). The degradation efficiency, determined from UV-vis absorption data after illumination, exhibited that the Mg–Mn-co-doped CdS photocatalyst degraded 85% of the dye in the absence of scavengers. After adding IPA, the degradation efficiency reduces to 42%, evidence of the ˙OH radicals being the dominant reactive species. BQ addition resulted in a moderate decrease to 68%, indicating a partial role of ˙O_2_^−^ radicals, whereas EDTA caused a relatively smaller decline to 77%, which suggests a minor contribution from holes (h^+^). So, the scavenger experiment ensures that hydroxyl radicals (˙OH) are the primary reactive species playing a role in pollutant degradation in the Mg–Mn-co-doped CdS photocatalytic system.

## 6. Antimicrobial activity

**Fig. 15:**
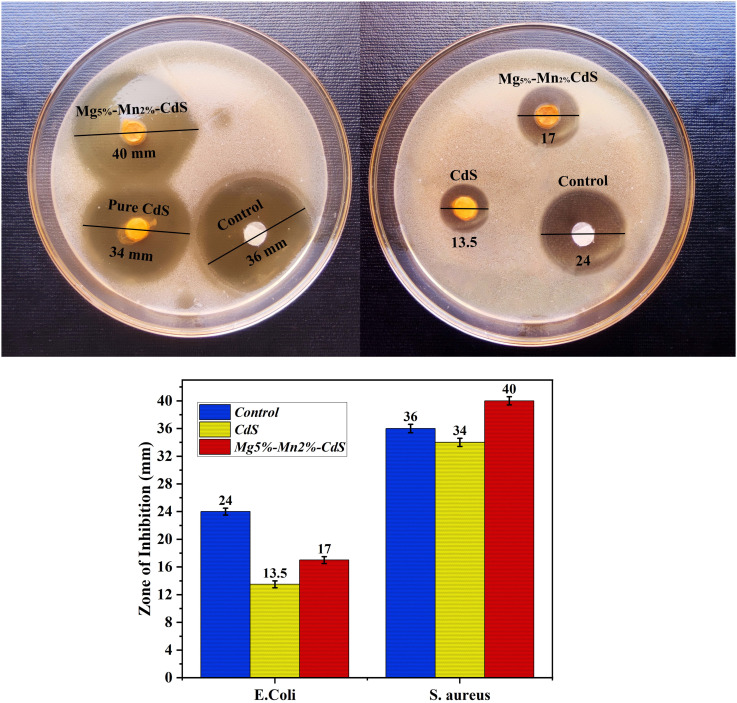
Comparative study of the antimicrobial activity of pure CdS, Mg_5%_–Mn_2%_-CdS and ampicillin, showing inhibition zone efficiency against *Escherichia coli* and *Staphylococcus aureus*.

This study expressed the antimicrobial properties of pure CdS and magnesium and manganese-co-doped cadmium sulfide (Mg_5%_–Mn_2%_-CdS) nanoparticles against *Escherichia coli* and *Staphylococcus aureus*, both of which are common pathogens with prominent clinical importance and values.^52^*E. coli* is one of the famous causes that may create gastrointestinal and urinary tract infections.^53^ On the other hand, *S. aureus* is also a cause for a variety of other infections, which include skin and wound infections, and sometimes, may cause more serious conditions like pneumonia as well as bloodstream infections.^54^ The basic goal was actually to assess the effectiveness of Mg_*x*_Mn_2%_CdS nanoparticles as antimicrobial agents and to compare their performance with that of pure CdS nanoparticles and the standard antibiotic, *i.e.*, ampicillin (Fig. 15).

The disk diffusion method was used to assess the antimicrobial activity of Mg_*x*_-Mn_2%_-CdS nanoparticles, which is mostly used on a large scale for evaluating the efficacy of antimicrobial agents.^55^ Sterile 6 mm disks were loaded with 20 µL of a 1 mg mL^−1^ Mg_5%_–Mn_2%_-CdS nanoparticle suspension, equivalent to 20 µg per disk. Then these disks were placed on LB agar plates, which were inoculated with the bacterial strains. The incubation of the plates was carried out at 37 °C for 24 hours.^56^ The next step was the measurement of the inhibition zones in millimetres. In light of comparative analysis, CdS nanoparticles and ampicillin was used as controls. All experiments were conducted in triplicate (*n* = 3), and results are presented as mean ± SD. One-way ANOVA was conducted, and the post-hoc test was conducted with Tukey. The level of significance was given as *p* < 0.05. The antimicrobial potential of Mg_5%_–Mn_2%_-CdS nanoparticles was clearly evidenced by the observed inhibition zones of *Escherichia coli*. The Mg_5%_–Mn_2%_-CdS nanoparticles showed excellent antimicrobial activity, with a zone of inhibition of 17 ± 0.2 mm (*p* < 0.05).

In comparison, CdS nanoparticles produced a zone of inhibition of 13.5 ± 0.3 mm (*p* < 0.05), which showed a relatively weaker antimicrobial effect. Ampicillin was used as the positive control, exhibiting a 24 ± 0.3 mm (*p* < 0.05) zone of inhibition, which was larger than that of the Mg_5%_–Mn_2%_-CdS nanoparticles.

Ampicillin was used as the positive control against *Staphylococcus aureus*, showing a zone of inhibition of 36 ± 0.2 mm (*p* < 0.05). Pure CdS exhibited an inhibition zone with a diameter of 34 ± 0.4 mm (*p* < 0.05). However, the Mg_5%_–Mn_2%_-CdS composite exhibited exceptional antibacterial activity, producing the largest zone of inhibition at 40 ± 0.3 mm (*p* < 0.05). These numerical findings are a clear indication that Mg and Mn-co-doped CdS nanoparticles exhibit a higher level of antibacterial activity compared to pure CdS. This enables the synergistic co-doping of Mn and Mg in the CdS matrix to effectively produce superoxide (˙O_2_^−^) and hydroxyl (OH˙) radicals that are important for destroying bacterial cell structures.^57^ These reactive species inactivate bacterial enzymes, thus interfering with the metabolism of the bacteria and eventually leading to cell death.^58^ Mg_5%_–Mn_2%_-CdS is the best sample because of its superior performance in terms of a higher ROS generation, which results in a disruption of bacteria. In contrast, pure CdS exhibits relatively weak antimicrobial behavior because of its larger bandgap and higher charge-carrier recombination rate.^59^ These characteristics restrict its ability to generate ROS effectively under similar experimental conditions.

The higher zones of inhibition shown by Mg_5%_–Mn_2%_-CdS nanoparticles compared to CdS and ampicillin makes it a very strong antimicrobial agent. The nanoparticles show high efficacy against diverse bacterial pathogens, including both Gram-negative bacterium (*e.g. E. coli*) and Gram-positive bacterium (*e.g. S. aureus*), which indicates their broad-spectrum antimicrobial capability. These results thus indicate that Mg–Mn-co-doped CdS nanoparticles might be promising materials for further development related to antimicrobial applications. The cytotoxicity and biocompatibility of Mg_5%_–Mn_2%_-CdS nanoparticles have not yet been evaluated experimentally. Therefore, biomedical applications at present are purely speculative and require additional data on their safety in mammals. To evaluate the practical relevance of the antibacterial performance, the activity of Mg–Mn-co-doped CdS was compared with previously reported CdS-based and other semiconductor nanomaterials (Table S4). While several materials exhibit larger inhibition zones or lower MIC values under optimized conditions, many of these systems require higher catalyst dosages, complex synthesis routes, or UV irradiation. In comparison, the present Mg/Mn-CdS system demonstrates competitive antibacterial activity under visible-light conditions using a low material dosage, highlighting its suitability for environmental and wastewater-related antibacterial applications rather than direct biomedical use. It should be noted that the antibacterial performance reported here is intended for environmental remediation contexts. Detailed biomedical evaluations, such as cytotoxicity and *in vivo* assessments, are beyond the scope of this study.

### 6.1 Mechanisms of antimicrobial action

The antimicrobial activity of CdS and Mag_5%_-Mn_2%_-CdS nanoparticles against bacteria cells is mainly due to the production of reactive oxygen species (ROS).^9^ When they interact with bacterial cells, they create an oxidative stress, causing the production of toxic reactive oxygen species (ROS), including hydroxyl radicals (˙OH), superoxide ions (˙O_2_^−^), and hydrogen peroxide (H_2_O_2_). The reactive oxygen species (ROS) react with DNA, proteins, and lipids in these bacteria, potentially destroying their cell membranes and eventually resulting in cell death.^60^

In addition to the production of reactive oxygen species (ROS), Mg_5%_–Mn_2%_-CdS nanoparticles damage bacterial cell membranes. The nanoparticles are positively charged, thus attracting the bacterial membranes possessing a negative charge. This results in the creation of pores in the bacterial membrane and thus leakage of intracellular components, which include ions, proteins, and nucleic acids, thus destroying the bacteria and limiting their survival capabilities.^61,62^

Moreover, ROS can hinder bacterial growth by disturbing the replication and transcription of DNA. The protein production is also affected by oxidative stress because of the disruption in the function of ribosomes and reduced efficiency of the enzymes.^63^

**Fig. 16:**
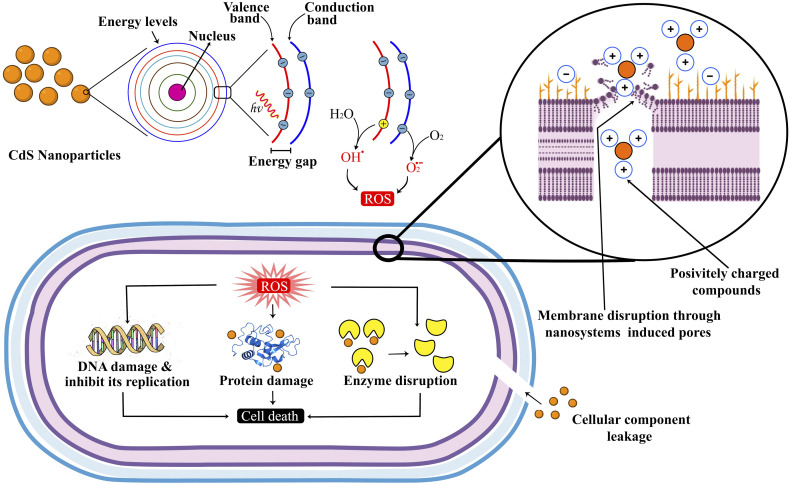
Schematic of the antimicrobial mechanism of Mg_5%_–Mn_2%_-CdS, illustrating the formation of electron–hole pairs and ROS and the disruption of bacterial cells.

The findings of this study confirm that Mg_5%_–Mn_2%_-CdS nanoparticles exhibit strong antimicrobial activity against both *Escherichia coli* and *Staphylococcus aureus*, outperforming CdS nanoparticles and showing efficacy comparable to ampicillin. The mechanism of action is mainly due to the production of highly reactive oxygen species (ROS), destabilization of bacterial cell membranes and inhibition of key bacterial processes like DNA replication and protein synthesis, as shown in Fig. 16. Due to such properties, they are promising in water purification, wound healing and pharmaceuticals. However, they should be examined more to determine their toxicity, stability over time, and clinical applicability.

## 7. Relative assessment of photocatalytic and antimicrobial activity

To further prove that this work is novel, the photocatalytic and antimicrobial performance of the Mg and Mn-co-doped CdS nanomaterial was compared to recently reported nanomaterial based on CdS. Under 125 min of visible-light irradiation (*k* = 0.014 min^−1^), the optimized Mg_5%_–Mn_2%_-CdS sample was able to degrade with a degradation efficiency of 84.79%, as shown in Table S4, which is better than the performance of most single- and co-doped CdS catalysts prepared by hydrothermal or solvothermal methods under similar conditions. Additionally, the co-doped CdS, which contained Mg and Mn, also demonstrated a high level of antibacterial activity against *Escherichia coli* and *Staphylococcus aureus*, which is a multidrug-resistant bacterium, thus proving the multifunctional capability. The improved performance is due to the synergistic effect of Mg^2+^ and Mn^2+^ ions, which is effective in enhancing charge-carrier separation and surface redox kinetics in the presence of visible-light illumination.

Compared with recently reported CdS-based photocatalysts, the Mg–Mn-co-doped CdS system exhibits competitive visible-light activity while using a relatively simple synthesis strategy for the preparation and a low dosage of the catalyst. Recent reports have revealed that more often than not, improved photocatalytic activities in CdS are either achieved by complex heterostructure formation or multi-step post-treatments.^64^

Conversely, the current paper underscores the fact that tuning lattice structure and electronic states with controlled co-doping of Mg^2+^ and Mn^2+^ results in inhibited radiative recombination and enhanced photocatalytic characteristics. Although the absolute rate of degradation is similar to some of the state-of-the-art systems, the findings highlight a structure–property law that can be used to rationalize the design of CdS-based photocatalysts in the future.^65^ It is important to mention that Table S4 deliberately incorporates reported photocatalysts with similar degradation efficiencies to those of the current system to prevent selective benchmarking. Nevertheless, the performance of photocatalysts differs considerably under a wide range of experimental conditions, including catalyst loading, light intensity, reactor configuration, and contaminant concentration, and therefore cannot be directly compared in a quantitative manner. Based on this, the comparison is made to illustrate relative material design strategies and performance trade-offs as opposed to making an absolute claim of superiority.

## 8. Conclusion

This work reports the successful synthesis and detailed characterization of CdS and Mg–Mn-co-doped CdS nanostructures, predicting enhanced photocatalytic and antimicrobial performance. Introducing defect states through co-doping with Mg and Mn results in the reduced bandgap, thus offering effective photocatalytic activity in the visible region of the spectrum and enhancing overall efficiency by improving charge-carrier separation. Mg_5%_–Mn_2%_-CdS was the optimal composition of the co-doped material, which offered the highest photocatalytic degradation efficiency of the dye at 84.79%, along with a reduction in PL intensity indicating lower recombination rates. SEM and XRD results depict the remarkable structural and morphological properties. Moreover, the material exhibited remarkable antibacterial activity against both Gram-positive and Gram-negative bacteria, compared to the pure CdS, also surpassing that of standard antibiotics. The cyclic stability and sustained activity of the catalyst over multiple cycles mark the potential for real-world applications in wastewater treatment and bacterial control. Overall, Mg–Mn-co-doped CdS is a promising multifunctional nanomaterial for environmental remediation and biomedical applications.

## Conflicts of interest

On behalf of all authors, the corresponding author states that there is no conflict of interest.

## Supplementary Material

RA-016-D6RA00066E-s001

## Data Availability

Data available at the following link: https://drive.google.com/drive/u/0/folders/1rdfCbhdAH406EcoxSx3pg4Qsi3-GphZY. Supplementary information (SI) is available. See DOI: https://doi.org/10.1039/d6ra00066e.
