# Supplementary material for: Mg/Mn co-doped CdS nanoparticles: a multifunctional platform for wastewater purification and antibacterial applications
Source: RSC Adv. 2026 Apr 21;16(23):20694–714. doi: 10.1039/d6ra00066e (PMC13098004; doi:10.1039/d6ra00066e)
Supplement: RA-016-D6RA00066E-s001 [file RA-016-D6RA00066E-s001.pdf]

Supplementary Information (SI) for RSC Advances.
This journal is © The Royal Society of Chemistry 2026

TABLE 1: The lattice parameters of pure CdS and **Mg-Mn Codoped CdS**

Sample	Structure	Lattice constants (Å)	Lattice angles (o)	Average Crystalline size (nm)	Calculated density (g/cm3)
**Pure CdS**	Hexagonal	a=b=4.136<br>c=6.713	α=β=90<br>γ =120	49.08	4.82
**$Mg_{1\%}$-$Mn_{2\%}$-CdS**	Hexagonal	a=b=4.139<br>c=6.717	α=β=90<br>γ =120	48.26	4.82
**$Mg_{3\%}$-$Mn_{2\%}$-CdS**	Hexagonal	a=b=4.14<br>c=6.719	α=β=90<br>γ =120	46.34	4.82
**$Mg_{5\%}$-$Mn_{2\%}$-CdS**	Hexagonal	a=b=4.144<br>c=6.722	α=β=90<br>γ =120	42.94	4.82
**$Mg_{7\%}$-$Mn_{2\%}$-CdS**	Hexagonal	a=b=4.146<br>c=6.726	α=β=90<br>γ =120	39.26	4.88

Table 2. Comparison of optical properties (band gap and PL intensity) and photocatalytic degradation activity of pure CdS and samples of Mg–Mn co-doped CdS

No	Catalyst	Bandgap eV	PL intensity (a.u.)	% Degradation
**1**	Pure CdS	2.8 eV	557	42
**2**	$Mg_{1\%}$-$Mn_{2\%}$-Cds	2.63 eV	416.39	54.66
**3**	$Mg_{3\%}$-$Mn_{2\%}$-Cds	2.53eV	310	68
**4**	$Mg_{5\%}$-$Mn_{2\%}$-Cds	2.19eV	203	85
**5**	$Mg_{7\%}$-$Mn_{2\%}$-Cds	2.37eV	253.5	77

Table 3. Shows the first order kinetic constant “k” and regression analysis for pure CdS and Mg and Mn Codoped CdS nanoparticles.

No	Photocatalysts	Rate constants “k” (min^{-1})	Correlation coefficients R^2
**1**	Pure CdS	0.0048	0.9866
**2**	$Mg_{1\%}$-$Mn_{2\%}$-CdS	0.00567	0.9793
**3**	$Mg_{3\%}$-$Mn_{2\%}$-CdS	0.00884	0.9812
**4**	$Mg_{5\%}$-$Mn_{2\%}$-CdS	0.01403	0.9804
**5**	$Mg_{7\%}$-$Mn_{2\%}$-CdS	0.00963	0.9853

Table S4. Comparison between photocatalytic and antimicrobial performance of CdS-based materials.

Sr No.	**Dopant System**	**Light Sourse & Conditions**	**Catalyst Loading/test**	**Photocatalytic Performance (k or % MB Removal**	**Antimicrobial Activity**	**References**
**1.**	Zn-Doped CdS	Solar Light,	1.5 g L^{-1}	88% degradation of MB	Not Reported	[49]
**2**	(CdS QDs)	Xe-lamps 100 W.	20 mg	80% 2-chlorophenol (2-CP)	both E. coli & A. hydrophila 20 mm	[64]
**3.**	Ni-doped $Cd_{0.9}Zn_{0.1}S$	Halogen lamp 300 W	0.4 g L^{-1}	88% degradation of MB	E. coli – 18 mm; S. aureus – 14 mm	[57]
**4.**	CdS-TiO2 nanocomposite	500 W Halogen light	1 g L^{-1}	84% degradation of Acid Blue-29	Not Reported	[65]
**5.**	Mn-doped CdS	Visible Light	1.0 g L^{-1}	~87% degradation of MB	Not Reported	[28]
**6.**	Mn-doped CdS	Blue LED (450 nm, 10 mW cm^{-2}	0.3 g L^{-1}	89% degradation of Tetracycline	Not reported	[66]
**7.**	Pure CdS (Our Work)	300 W Xenon Lamp	(0.5, 1.0, 1.5) g L^{-1}	36%, 42%, 47%	E. coli: 13.5 ± 0.3, S. aureus: 34 ± 0.4	This Work
**8.**	$Mg_{5\%}$-$Mn_{2\%}$-CdS	300 W Xenon Lamp	(0.5, 1.0, 1.5) g L^{-1}	(81%, 84.79%, 89%	E. coli: 17 ± 0.2, S. aureus: 40 ± 0.3	This Work
